# Trends in CD4 counts in HIV-infected patients with HIV viral load monitoring while on combination antiretroviral treatment: results from The TREAT Asia HIV Observational Database

**DOI:** 10.1186/1471-2334-10-361

**Published:** 2010-12-23

**Authors:** Jialun Zhou, Thira Sirisanthana, Sasisopin Kiertiburanakul, Yi-Ming A Chen, Ning Han, Poh_Lian Lim, Nagalingeswaran Kumarasamy, Jun Yong Choi, Tuti Parwati Merati, Evy Yunihastuti, Shinichi Oka, Adeeba Kamarulzaman, Praphan Phanuphak, Christopher KC Lee, Patrick CK Li, Sanjay Pujari, Vanthanak Saphonn, Matthew G Law

**Affiliations:** 1 National Centre in HIV Epidemiology and Clinical Research, The University of New South Wales, Sydney, Australia; 2 Research Institute for Health Sciences, Chiang Mai University, Chiang Mai, Thailand; 3 Faculty of Medicine Ramathibodi Hospital, Mahidol University, Bangkok, Thailand; 4 Taipei Veterans General Hospital and AIDS Prevention and Research Centre, National Yang-Ming University, Taipei, Taiwan; 5 Beijing Ditan Hospital, Beijing, China; 6 Tan Tock Seng Hospital, Singapore; 7 YRG Centre for AIDS Research and Education, Chennai, India; 8 Department of Internal Medicine and AIDS Research Institute, Yonsei University College of Medicine, Seoul, Korea; 9 School of Medicine Udayana University & Sanglah Hospital, Denpasar, Bali, Indonesia; 10 Working Group on AIDS Faculty of Medicine, University of Indonesia/Ciptomangunkusumo Hospital, Jakarta, Indonesia; 11 International Medical Centre of Japan, Tokyo, Japan; 12 University of Malaya Medical Centre, Kuala Lumpur, Malaysia; 13 HIV-NAT/Thai Red Cross AIDS Research Centre, Bangkok, Thailand; 14 Hospital Sungai Buloh, Kuala Lumpur, Malaysia; 15 Queen Elizabeth Hospital, Hong Kong, China; 16 Institute of Infectious Diseases, Pune, India; 17 National Center for HIV/AIDS, Dermatology & STDs, Phnom Penh, Cambodia

## Abstract

**Background:**

The aim of this study was to examine the relationship between trends in CD4 counts (slope) and HIV viral load (VL) after initiation of combination antiretroviral treatment (cART) in Asian patients in The TREAT Asia HIV Observational Database (TAHOD).

**Methods:**

Treatment-naive HIV-infected patients who started cART with three or more and had three or more CD4 count and HIV VL tests were included. CD4 count slopes were expressed as changes of cells per microliter per year. Predictors of CD4 count slopes from 6 months after initiation were assessed by random-effects linear regression models.

**Results:**

A total of 1676 patients (74% male) were included. The median time on cART was 4.2 years (IQR 2.5-5.8 years). In the final model, CD4 count slope was associated with age, concurrent HIV VL and CD4 count, disease stage, hepatitis B or C co-infection, and time since cART initiation. CD4 count continues to increase with HIV VL up to 20 000 copies/mL during 6-12 months after cART initiation. However, the HIV VL has to be controlled below 5 000, 4 000 and 500 copies/mL for the CD4 count slope to remain above 20 cells/microliter per year during 12-18, 18-24, and beyond 24 months after cART initiation.

**Conclusions:**

After cART initiation, CD4 counts continued to increase even when the concurrent HIV VL was detectable. However, HIV VL needed to be controlled at a lower level to maintain a positive CD4 count slope when cART continues. The effect on long-term outcomes through the possible development of HIV drug resistance remains uncertain.

## Background

Studies show that latent infection of CD4 cells provides a mechanism for lifelong persistence of HIV-1, even in patients on effective anti-retroviral therapy [1]. To suppress viral replication so that the VL is below the level of detection with standard assays is thus one of the aims at the start of antiretroviral treatment. Maximal and durable suppression of HIV VL prevents or delays development of drug resistant mutations, preserves CD4 cells, and eventually results in better clinical outcomes. According to the US guidelines, if HIV VL suppression is not achieved, it is necessary to change to a new regimen, a second or third line regimen, with at least two active drugs [2].

HIV-infected patients in most developing countries have limited second and third line antiretroviral treatment options [3]. In many countries in Asia, second-line combination antiretroviral treatment (cART) is not widely accessible [4-6]. There remains some uncertainty about the short-term risks to patients receiving first line cART, in particular how their immune status might deteriorate if they persist with a virologically failing regimen. The Pursuing Later Treatment Options (PLATO) collaboration [7] reported that in patients experiencing triple class failure, treatment regimens that maintain the VL below 10 000 copies/mL or at least provide 1.5 log10 copies/mL suppression below the off-treatment value do not seem to be associated with appreciable CD4-cell-count decline. More recently, Mocroft et al [8] also reported that CD4 did not significantly decrease even HIV VL exceeded 10 000 copies/mL in patients treated with regimen containing a boosted protease inhibitor. The issue of when to switch from first line regimens may therefore be difficult, especially for patients with modest, stable HIV VL who are clinically doing well [5,9].

The aims of this study were to examine the relationship between trends in CD4 count and VL after initiation of combination antiretroviral treatment in HIV-infected Asian patients, using data from The TREAT Asia HIV Observational Database (TAHOD).

## Methods

Established in 2003, TAHOD is a collaborative observational cohort study involving 18 sites in the Asia-Pacific region (See acknowledgement). Detailed methods are published elsewhere [10]. Briefly, each site recruited approximately 200-300 HIV-infected patients, including both patients on or not initiating antiretroviral treatment. Recruitment was based on a consecutive series of patients regularly attending a given site from a particular start-up time. Ethical approval for the study was obtained from the University of New South Wales Ethics Committee and respective local ethics committee.

The following data were collected: patient demographics and baseline data, CD4 and CD8 count, HIV VL level, prior and new AIDS defining illness (ADI), date and cause of death, prior and current prescribed HAART, and reason for treatment change. Data are collected according to a common protocol. Upon recruitment, all available data prior to entry to TAHOD (considered as retrospective data) are extracted from patient case notes. Prospective data are updated six-monthly at each clinic and transferred to data management centre for aggregation and analyses. TAHOD sites are encouraged to contact patients who were not seen in the clinics in the previous 12 months.

TAHOD patients were included in this analysis if they were treatment naïve and initiated with triple or more combination antiretroviral treatment, and had three or more concurrent CD4 and HIV VL test pairs (within 28 days if not tested on the same day) during follow-up. Both retrospective and prospective data were included in the analysis.

Trends in CD4 count (slope) was calculated by linear regression with the values at time T, before T, and after T, and was expressed as changes of cells per microliter (μL) per year. The HIV VL was related to the CD4 count slope at time T. Previous studies reported a two-phase CD4 count response, demonstrated as a rapid increase (a high CD4 count slope) in the first several months after treatment initiation and followed by slower increase (a smaller slope compared to the initial several months)[11-14]. Preliminary analyses in eligible TAHOD patients showed that the mean CD4 count slope was significantly higher in the first 6 months after cART initiation than in the period afterwards (179 vs. 44 cells/μL per year, p < 0.001). The CD4 slopes were therefore calculated from CD4 counts measured 6 months after cART initiation.

Predictors of the CD4 slope at time T was assessed by random-effect linear regression models which take account of within and between patient variability. Covariates included sex, age (per 10 years), disease stage (CDC category A, tuberculosis with or without other AIDS defining illness, other non-tuberculosis AIDS defining illness), hepatitis coinfection (hepatitis B or C antibody positive), haemoglobin level, time since cART initiation, initial cART regimen non-nucleoside/nucleotide reverse transcriptase inhibitor (NNRTI)-based, non-boosted PI, or boosted PI, and treatment containing abacavir. Disease stage, CD4 count and HIV VL were fitted in the model as time-dependent variable. We did not include CD4 count and HIV VL at baseline for the following three reasons: first, a large proportion of patients did not have the tests at treatment initiation (approximately 25% of patients had no CD4 count and 45% HIV VL, Table 1); second, the model aimed to help clinicians in this region to assess the status of immune system with the clinical information at hand (e.g., age, hepatitis status, current CD4 count, time since treatment initiation, etc) where the baseline information on CD4 count and HIV VL may not be readily available; and third, when we included baseline CD4 and HIV VL in a sensitivity analyses based on the subset of patients with baseline data available, the results remained comparable with the model without the baseline CD4 count and HIV VL. The multivariate models were built using a forward-step approach, the final model included covariates that remained significant at the 0.20 level. Non-significant variables were also presented and adjusted for in the final multivariate models.

**Table 1:** Patient characteristics at baseline in patients selected in the analysis and patients starting 3 or more cART in TAHOD

	In analysis	In TAHOD		In analysis	In TAHOD
Total patients	1676	4056	Haemoglobin (g/dL) at cART initiation		
Age (year) at cART initiation			Median (IQR)	14.0 (12.6, 15.2)	12.3 (10.8, 14.2)
Median (IQR)	36 (30, 42)	35 (30, 41)	< 10 g/dL	27 (2%)	418 (16%)
<=30	437 (26%)	1188 (29%)	10+g/dL	1248 (98%)	2250 (84%)
31~40	748 (45%)	1814 (45%)	Not tested	221	1388
41 or more	491 (29%)	1054 (26%)	CD4 count (cells/μL) at cART initiation		
			Median (IQR)	140 (42, 230)	112 (37, 209)
Gender			<=50	350 (28%)	990 (31%)
Male	1238 (74%)	2875 (71%)	51-100	155 (12%)	508 (16%)
Female	438 (26%)	1177 (29%)	101-200	328 (26%)	815 (26%)
Transgender	0 (0%)	4 (< 1%)	201-300	261 (21%)	551 (17%)
			301 or more	161 (13%)	311 (10%)
CDC clinical classification for HIV infection at cART initiation			Not tested	421	881
Stage A	980 (58%)	1984 (50%)	HIV viral load (copies/ml) at cART initiation		
Stage B	101 (6%)	407 (10%)	Median log10 (IQR)	4.93 (4.22, 5.52)	4.94 (4.32, 5.51)
Stage C	595 (36%)	1665 (41%)	< 500	101 (11%)	126 (9%)
			501-10,000	80 (9%)	124 (9%)
Hepatitis B or C coinfection			10,001-50,000	173 (19%)	280 (20%)
No	1468 (88%)	3495 (86%)	50,001 or more	555 (61%)	885 (62%)
Yes	208 (12%)	566 (14%)	Not tested	767	2641

To take into consideration of the treatment interruption and switch, the following sensitivity analyses were performed: 1. restricting the records measured during initial NNRTI-based regimen; 2. excluding the records measured when patients were off-treatment for more than 30 days for various reasons. Finally, sensitivity analysis was also performed by restricting the records in patients contributing at least 4 or more concurrent CD4 and HIV VL tests.

Data management and statistical analyses were performed using SAS for Windows (SAS Institute Inc., Cary, NC, USA), and Stata (StataCorp, STATA 10.1 for Windows, College Station, Texas 77845 USA).

## Results

There were 4699 patients with data collected in TAHOD as at September 2009. Approximately 75% of patients had a clinic visit in the 12 months before September 2009, and 214 patients died since entry to TAHOD (mortality 1.36 per 100 person years). Among the 4699 TAHOD patients, 612 were not currently receiving antiretroviral treatment, 31 were receiving mono or dual therapy, and 4056 had initiated cART with three or more drugs. 1676 naïve patients initiated cART, and had three or more concurrent CD4 and HIV VL data pairs available beyond 6 months after cART initiation.

Table 1 shows the patient characteristics at cART initiation in patients included in the analysis and in all TAHOD patients who initiated cART with three or more drugs. The characteristics of the patients included in the analysis are generally comparable to those of the whole TAHOD patients, except that the patients included were less likely to be anemic.

At cART initiation, the median age of the patients included in the analysis was 36 years (interquartile range, IQR, 30-42), median CD4 count 140 cells/μL (IQR 42-230), median HIV VL 5.00 log10 copies/mL (IQR 4.33-5.56), 12% had hepatitis B or C co-infection, and 36% were diagnosed with an AIDS defining illness (ADI). The median time on cART was 4.2 years (IQR 2.5 - 5.8). The median time between each CD4 and HIV VL tests was 165 days (IQR 106 - 223). The initial cART was predominantly an NNRTI-based regimen (63% of 1676 patients in the analysis, with either nevirapine or efavirenz, plus two NRTI drugs, mostly stavudine or zidovudine, plus lamivudine). Approximately 15% of the patients started a non-boosted PI (mostly indinavir, nelfinavir or atanazavir) regimen with two NRTI drugs and 20% started with a ritonavir-boosted PI (mostly lopinavir, atanazavir or saquinavir). The annual rate of a drug class change or change of at least two or more drugs was approximately 20%. After cART initiation, viral logical suppression (HIV VL < 400 copies/mL) was achieved in 83% of patients at 6 month and 82% in 12 months.

Table 2 shows the random-effect linear regression analysis of the CD4 count slope. Concurrent haemoglobin level, initial cART containing NNRTI or boosted PI were not significantly associated with the study endpoint in both univariate and multivariate analyses. Initial cART containing abacavir was significant in the univariate analysis. In the final multivariate model, CD4 count slope was associated with age (-4.8 cells/μL per year per 10-year age increase, p = 0.013), concurrent HIV VL (-40.5 per 1 log10 copies/mL VL increase, p < 0.001), concurrent CD4 count (+1.9 per 100 cells/μL increase), disease stage (compared to CDC category A illnesses: +26.3 if diagnosed with tuberculosis [TB] with or without other ADI, p < 0.001; +12.0 if diagnosed with non-TB ADI, p = 0.013), hepatitis B or C co-infection (-17.7 if co-infected, p = 0.004), and time since cART initiation (compared to CD4 slope during 6-12 months: -21.5 during 12-18 months, p = 0.010; -25.8 during 18-24 months, p = 0.002; -59.1 at 24 months or later, p < 0.001).

**Table 2:** Random-effect linear regression analyses of trend of CD4 count (slope, cells/μL per year)

	Univariate			Multivariate**		
	Difference*	(95% CI)	p value	Difference*	(95% CI)	p value
Sex						
Male*	0.0			0.0		
Female	9.5	(0.1, 18.9)	0.047	7.8	(-1.5, 17.2)	0.099
Current age						
per 10 years older	-6.5	(-10.3, -2.7)	0.001	-4.8	(-8.6, -1.0)	0.013
Disease stage						
CDC Category A*	0.0			0.0		
TB with or without other ADI	24.4	(13.7, 35.1)	< 0.001	26.3	(15.6, 37.0)	< 0.001
Non-TB ADI(s)	3.7	(-5.6, 13.0)	0.433	12.0	(2.5, 21.4)	0.013
Haemglobin level						
per 1 g/dL higher	0.0	(-0.0, 0.1)	0.661	0.0	(-0.0, 0.1)	0.689
Concurrent CD4 count						
Per 100 cells/μL higher	1.3	(-0.3, 3.0)	0.116	1.9	(0.2, 3.7)	0.033
Concurrent viral load						
per log10 copies/mL higher	-40.9	(-48.7, -33.2)	< 0.001	-40.5	(-48.4, -32.6)	< 0.001
Hepatitis B or C coinfection						
No*	0			0		
Yes	-19.1	(-31.1, -7.0)	0.002	-17.7	(-29.7, -5.7)	0.004
Time since cART initiation						
> 6 to ≤ 12 months*	0.0			0.0		
> 12 to ≤ 18 months	-21.3	(-37.7, -5.0)	0.011	-21.5	(-37.8, -5.2)	0.010
> 18 to ≤ 24 months	-23.1	(-39.3, -7.0)	0.005	-25.8	(-42.0, -9.7)	0.002
> 24 or more months	-58.0	(-70.3, -45.7)	< 0.001	-59.1	(-71.7, -46.5)	< 0.001
Initial cART containing NNRTI						
No*	0.0			0.0		
Yes	6.4	(-1.4, 14.1)	0.106	-1.3	(-9.2, 6.6)	0.749
Initial cART containing boosted PI						
No*	0.0			0.0		
Yes	-0.8	(-10.5, 9.0)	0.880	-3.4	(-13.2, 6.3)	0.493
Initial cART containing abacavir						
No*	0.0					
Yes	-12.9	(-25.8, 0.0)	0.050	-5.6	(-18.5, 7.3)	0.397

* Difference were compared with the first category of each categorical variable.

** Constant term for multivariate model: 205.5 (174.4, 236.6) cells per μL per year.

**Figure 1:**
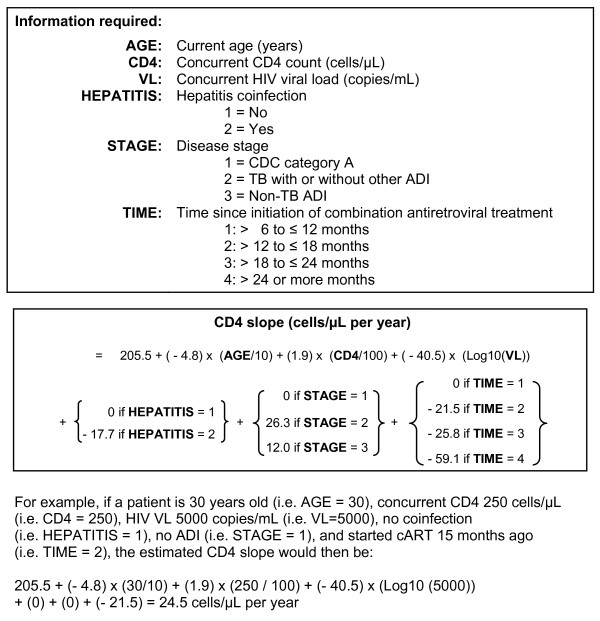
**Estimating CD4 count slope**.

In Figure 1 we gave the formula obtained from Table 2 and an example to estimate the CD4 slope. The formula needs the following information: current age, concurrent CD4 count and HIV VL, hepatitis coinfection, disease stage, and time since cART initiation.

**Figure 2:**
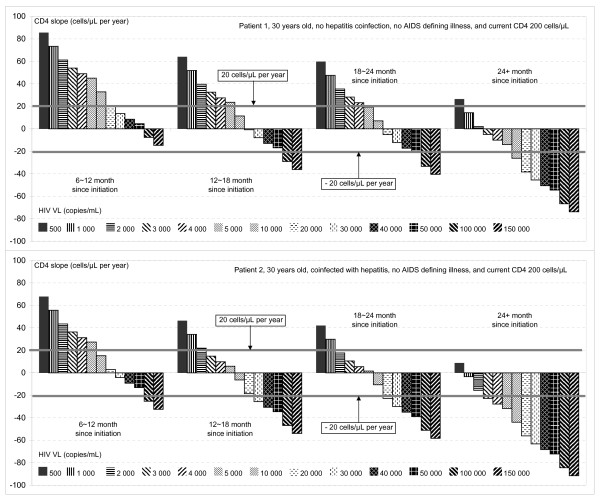
**Estimated CD4 slope (cells/μL/year) by duration of treatment and HIV VL**.

The models shows that, after cART initiation, mean CD4 counts continued to increase even when the concurrent HIV VL was detectable. In addition, the model also shows that to maintain a positive CD4 count slope, the HIV VL needed to be suppressed to a lower level in later periods of cART. To illustrate, the estimated CD4 slopes from the model in two patients with specific baseline characteristics are shown in Table 3 and in Figure 2. The bold cells are when the estimated CD4 count slope falls between -20 and +20 cell/μL per year, which we considered as indicative of borderline CD4 count decreases. In the case of the first patient (aged 30 years, no hepatitis coinfection and AIDS defining illness, and concurrent CD4 count 200 cells/μL), the CD4 count continues to increase with HIV VL up to 20 000 copies/mL during 6-12 months after cART initiation. However, the HIV VL has to be controlled below 5 000, 4 000 and 500 copies/mL for the CD4 count slope to remain on a safe level above 20 cells/μL/year during 12-18, 18-24, and beyond 24 months after cART initiation.

**Table 3:** Estimated CD4 slope (cells/μL/year) by duration of treatment and HIV VL.

Month since cART initiation	HIV VL level (copies/mL)												
	500	1 000	2 000	3 000	4 000	5 000	10 000	20 000	30 000	40 000	50 000	100 000	150 000
	Patient 1, 30 years old, no hepatitis coinfection, no AIDS defining illness, and current CD4 200 cells per μL												
6-12	85.6	73.4	61.2	54.1	49.0	45.1	32.9	20.7	**13.6**	**8.5**	**4.6**	**-7.6**	-14.7
12-18	64.1	51.9	39.7	32.6	27.5	23.6	**11.4**	**-0.8**	**-7.9**	**-13.0**	**-16.9**	-29.1	-36.2
18-24	59.8	47.6	35.4	28.3	23.2	**19.3**	**7.1**	**-5.1**	**-12.2**	**-17.3**	-21.2	-33.4	-40.5
24+	26.5	**14.3**	**2.1**	**-5.0**	**-10.1**	**-14.0**	-26.2	-38.4	-45.5	-50.6	-54.5	-66.7	-73.8
													
	Patient 2, 30 years old, coinfected with hepatitis, no AIDS defining illness, and current CD4 200 cells per μL												
6-12	67.9	55.7	43.5	36.4	31.3	27.4	**15.2**	**3.0**	**-4.1**	**-9.2**	**-13.1**	-25.3	-32.4
12-18	46.4	34.2	22.0	**14.9**	**9.8**	**5.9**	**-6.3**	**-18.5**	-25.6	-30.7	-34.6	-46.8	-53.9
18-24	42.1	29.9	**17.7**	**10.6**	**5.5**	**1.6**	**-10.6**	-22.8	-29.9	-35.0	-38.9	-51.1	-58.2
24+	**8.8**	**-3.4**	**-15.6**	-22.7	-27.8	-31.7	-43.9	-56.1	-63.2	-68.3	-72.2	-84.4	-91.5

Hepatitis co-infection had a significant effect on the CD4 count slope. In one scenario, shown in Table 3 of a 30-year old patient with concurrent CD4 count 200 cells/μL, no AIDS defining illness and no hepatitis coinfection, CD4 counts continues to increase with HIV VL up to 5 000 copies/mL during 12-18 months after cART. If this patient was hepatitis co-infected, the CD4 count starts to fall when the HIV VL increases up to 3 000 copies/mL.

The analyses were repeated in three subgroups as sensitivity analysis (Table 4). The results are comparable to the final model.

**Table 4:** Sensitivity analyses of the CD4 count slope.

	Final model		Initial treatment, before any class change or stop for more than 30 days		Initial NNRTI-based regimen, before change or stop for more than 30 days		Including patients with 4 or more CD4 slopes endpoints	
	
	Difference*	(95% CI)	Difference*	(95% CI)	Difference*	(95% CI)	Difference*	(95% CI)
		Observations = 10899		Observations = 6697		Observations = 4058		Observations = 9826
		Patients = 1676		Patients = 1353		Patients = 863		Patients = 1079
Current age, per 10 yrs older								
per unit	-4.8	(-8.6, -1.0)	-4.0	(-9.0, 1.0)	-2.4	(-9.2, 4.4)	-4.3	(-8.4, -0.2)
Concurrent viral load, per log10 copies per mL higher								
per unit	-40.5	(-48.4, -32.6)	-43.4	(-58.5, -28.4)	-45.1	(-68.0, -22.2)	-39.5	(-48.1, -30.9)
Concurrent CD4 per 100 per μL higher								
per unit	1.9	(0.2, 3.7)	3.4	(1.0, 5.7)	4.1	(0.9, 7.3)	1.9	(0.0, 3.8)
Disease stage								
CDC Category A*	0.0		0.0		0.0		0.0	
TB and/or other ADI	26.3	(15.6, 37.0)	29.9	(16.1, 43.7)	29.9	(13.9, 45.9)	26.4	(14.6, 38.1)
Non-TB ADI(s)	12.0	(2.5, 21.4)	16.4	(3.4, 29.4)	16.3	(-1.2, 33.9)	11.2	(0.9, 21.6)
Hepatitis B or C coinfection								
No*	0.0		0.0		0.0		0.0	
Yes	-17.7	(-29.7, -5.7)	-17.5	(-33.1, -2.0)	-19.8	(-39.6, 0.0)	-18.7	(-31.9, -5.5)
Time since ART initiation								
> 6 to ≤ 12 months*	0.0		0.0		0.0		0.0	
> 12 to ≤ 18 months	-21.5	(-37.8, -5.2)	-29.4	(-48.2, -10.6)	-16.3	(-40.9, 8.3)	-19.6	(-37.8, -1.4)
> 18 to ≤ 24 months	-25.8	(-42.0, -9.7)	-32.8	(-51.9, -13.7)	-13.9	(-38.8, 10.9)	-25.8	(-43.6, -7.9)
> 24 or more months	-59.1	(-71.7, -46.5)	-65.6	(-80.7, -50.5)	-55.4	(-75.3, -35.5)	-59.1	(-73.1, -45.0)
Constant	205.5	(174.4, 236.6)	207.2	(157.7, 256.6)	192.8	(121.4, 264.2)	200.6	(166.6, 234.6)

* Difference were compared with the first category of each categorical variable

## Discussion

In a subset of TAHOD patients who were treatment naïve and initiated with three or more combination antiretroviral treatment and had concurrent CD4 count and HIV VL tests, the CD4 count slope was associated with age, concurrent CD4 count and HIV VL, disease stage, hepatitis coinfection and time since cART initiation. After cART initiation, CD4 counts continued to increase even when the concurrent HIV VL was detectable. However, HIV VL needed to be controlled at a lower level to maintain a positive CD4 count slope when cART continues at later stages, particularly from 6 months to more than 24 months after cART initiation.

The inverse relationship between age and CD4 restoration has been reported in previous studies. In these studies younger age was associated with more rapid CD4 recovery and was associated with preserved thymic function [7,13-15]. The increase in CD4 slope after TB diagnosis, compared to CDC category A illness, might seem counterintuitive. This might be simply due to the increased total lymphocytes during active infections. The increase could also be the short-term response due to the increased adherence to cART [16,17] and introduction of treatment for TB or other ADI [18,19].

Studies have shown that neither HBV nor HCV coinfection influence virological response to cART [20-22]. However, in terms of immunological response, the results were mixed [20,21,23-25]. Law et al observed in HIV-infected patients with HBV or HCV an initially delayed CD4 count recovery at week four after HAART treatment, but at week 48 the CD4 count increase was similar to the patients only infected with HIV [26]. These studies examine the absolute CD4 count rather than the trend since cART initiation. A decrease in CD4 count slope of less than 20 cells might not be clinically significant in the early phase of cART, but from our estimates (Table 3), it does have a significant impact on whether the CD4 count slope decreased after longer durations of cART. For example, the patient with no hepatitis co-infection would continue to have a CD4 count increase over 20 cells/μL more than 24 months after cART initiation even the concurrent HIV VL is above 500 copies/mL. If this patient is co-infected with hepatitis and on cART for more than 24 months, the CD4 count slope is below 20 cells/μL even the concurrent HIV VL is 500 copies/mL.

The PLATO study [7] reported that in patients experiencing triple class failure, treatment regimens that maintain the VL below 10 000 copies/mL or at least provide 1.5 log10 copies/mL suppression below the off-treatment value do not seem to be associated with appreciable CD4-cell-count decline. In a combined analysis between Asian and Australian patients infected HIV, Egger et al [14] reported a three-way interaction between the time since cART, baseline CD4 and post-cART HIV VL and estimated that for patients with intermittent HIV viral suppression (below 400 copies/mL), the mean absolute CD4 count begins to decrease or plateau after 4 years of cART. These studies and our findings show that after cART initiation, mean CD4 count slope can continued to increase even when the concurrent HIV VL is detectable. While Egger et al introduced the effect of time in the equation of long term patterns of CD4 response, the results from this analysis further added that the concurrent HIV VL level is a significant factor in determining the trend of CD4 after cART.

Using data from EuroSIDA, Mocroft et al [8] reported that CD4 did not significantly decrease even HIV VL exceeded 10 000 copies/mL in patients treated with regimen containing a boosted protease inhibitor. Drug class and cART containing abacavir was also included in the analysis, however, none remained significant in the final model. This might be due to three reasons: first, the paper by Mocroft et al analysed data from EuroSIDA where the predominant cART regimen was PI-based (46% non-boosted, 23% boosted PI). TAHOD recruits patients from the Asia Pacific region, with NNRTI-based regimen as the most common initial cART (63%, 15% non-boosted and 20% boosted PI). In addition, abacavir was not frequently used in TAHOD; second, the patients who received PI- or NNRTI-based cART as initial regimen might be different between EuroSIDA and TAHOD, which could result in a different recovery pattern of the immune system; three, as suggested by Mocroft et al, larger studies with increased power are needed. Nonetheless, our study provided complementary evidence in patients from Asia Pacific region that CD4 counts continues to increase even when the concurrent HIV VL was detectable.

Similar to other studies [11-13], our data showed a two-phase CD4 count response with a high CD4 count slope in the first six months after treatment initiation followed by a lower slope. The only factor in the final multivariate model (Table 2) that could be modified and had a significant impact on CD4 count slope was the concurrent HIV VL, which is a 40 CD4 cells decrease for every 1 log10 HIV VL increase. From our estimation (Table 3), the CD4 count continues to increase with HIV VL up to 20 000 copies/mL during 6-12 months after cART initiation. However, the HIV VL has to be controlled below 5 000, 4 000 and 500 copies/mL for the CD4 count slope to reach a safe level above 20 cells/μL/year during 12-18, 18-24, and beyond 24 months after cART initiation.

In many countries in Asia, second-line cART is not widely accessible [3-6]. Several studies reported sustainable CD4 count increases in patients with virological failure but remained on the same failing cART [27-29]. Our results suggest that patients with detectable but modest VL may continue their failing cART regimen without increasing their immune deficiency and the risk of poor clinical outcomes over the short term. This is in agreement with the US treatment guideline [2], which recommended adherence assessment, repeated HIV VL tests to rule out "blips" [30], and genotypic tests to detect drug resistant mutations before considering treatment switch. The recent 2009 revision of the WHO antiretroviral therapy guidelines [31] recommended adherence assessment, repeated HIV VL test, and switch only when HIV VL remains more than 5 000 copies/mL. If HIV VL monitoring is available, switch to second-line cART should be done as soon as possible when treatment failure is established. However, in many countries in Asia, especially those developing countries, frequent HIV VL monitoring and genotypic tests are beyond the limited resource for HIV treatment and care [32]. If CD4 count is the only way for monitoring treatment response, the result of this analysis showed that a patient can have a considerable duration of virological failure without meeting CD4 criteria recommended by WHO for switch of ART to second line. In addition, the effect of delaying switching treatment on longer term outcomes through the possible development of HIV-drug resistance that could compromise the efficacy of later cART regimens remains uncertain.

## Limitations

Several limitations should be considered in interpreting the results in this paper. First, TAHOD participating sites are generally urban referral centres, and each site recruits 200-300 patients who are considered by local clinicians to have a reasonably good prospect of long-term follow-up. Hence TAHOD patients, and their treatment, are not representative of all HIV-infected patients in the Asia and Pacific region. Second, we do not have data on adherence and treatment against TB and other ADI. Finally, a more thorough analysis would include the survival outcome. However, because of the limited number and follow-up of patients who were failing virologically, this analysis is currently underpowered. Further analyses will be considered with longer duration of follow-up.

## Conclusion

The analyses suggest that after cART initiation, mean CD4 slope can continue to increase even when the concurrent HIV VL is detectable. HIV VL needed to be controlled at a lower level to maintain a positive CD4 slope beyond 2 years of cART. However, the effect on longer term outcomes through the possible development of HIV-drug resistance remains uncertain.

## Competing interests

The authors declare that they have no competing interests.

## Authors' contributions

JZ and ML originated the study concept and detailed the analysis plan. JZ performed the data manipulation, statistical analysis, interpretation of results and drafted the manuscript. TS, SK, YMC, NH, PLL, NK, JYC, TPT and EY commented on the study concept and analysis plan, helped interpretation of results and edited the manuscript. SO, AK, PP, CL, PL, VS reviewed the manuscript and provided clinical interpretations. All authors read and approved the final manuscript.

## Pre-publication history

The pre-publication history for this paper can be accessed here:

http://www.biomedcentral.com/1471-2334/10/361/prepub
